# Concrete400: A multi-task pixel-level crack dataset from urban infrastructure in Vietnam

**DOI:** 10.1016/j.dib.2026.113246

**Published:** 2026-09-09

**Authors:** Le Hoai Nam, Nguyen Ngoc Lan, Vu Manh Trung, Nguyen Nam Son, Vu Quang Tuyen, Tran Ngoc Hoa

**Affiliations:** a Application of Artificial Intelligence for Structural Health Monitoring (AI-SHM) Research Group, Faculty of Civil and Environmental Engineering (R2026-UTC-01), University of Transport and Communications, Hanoi, Vietnam; b Office of International Education Programs, University of Transport and Communications, Hanoi, Vietnam

**Keywords:** Concrete crack dataset, Classification, Image segmentation, Object detection, Infrastructure inspection

## Abstract

Concrete400 is a multi-task concrete crack dataset for visual infrastructure inspection. It contains 379 RGB photographs of building surfaces and concrete pavements in Hanoi, Vietnam, with manually prepared pixel-level crack masks and corresponding text files containing crack region bounding box annotations. The release also includes 1502 labelled image patches for binary crack classification, comprising 633 crack patches and 869 no-crack patches. The collection preserves field variability in illumination, concrete texture, surface condition, background clutter, and acquisition time. The released segmentation and object detection images, masks, and box coordinates are standardised to 512 × 512 pixels, while the classification patches are provided at 256 × 256 pixels. The accompanying reproducible protocol defines 303 training and 76 validation image–mask pairs. After masks are binarised at an intensity threshold of 127, crack pixels represent 3.08% of image pixels on average. Concrete400 can therefore support patch-level crack classification, semantic crack segmentation, instance-style crack region segmentation, object detection, semi-supervised and self-supervised learning, and crack image generation under realistic inspection conditions.

Specifications Table

**Table:** 

Subject	Computer Science; Civil Engineering; Materials Science.
Specific subject area	Computer Vision, Classification, Semantic and Instance-style segmentation, Object detection, Automated visual inspection of concrete infrastructure.
Type of data	RGB images, binary crack masks, crack region bounding box annotation files, bounding box visualisations, and binary crack/no-crack image patches.
How the data were acquired	High-resolution smartphone photographs were acquired from concrete building surfaces and pavements in Hanoi, Vietnam. Pixel-level crack masks were prepared manually in Adobe Photoshop.
Data format	Standardised 512×512 JPEG image–mask pairs, TXT bounding box annotation files, PNG bounding box visualisations, and 256 × 256 JPEG classification patches.
Description of data collection	The collection retains natural variations in illumination, concrete texture, surface state, background clutter, and acquisition time.
Data source location	Building surfaces and concrete pavements, Hanoi, Vietnam.
Data accessibility	Repository name: Zenodo Data identification number: 10.5281/zenodo.21193945 Direct URL to data: https://zenodo.org/records/21193945
Related research article	None

## 1 Value of the Data

• **Real-world Environmental Realism**: Unlike laboratory controlled collections or pavement datasets built for highways, Concrete400 captures the complex and diverse environmental conditions of active urban concrete infrastructure in Hanoi, Vietnam. It features severe illumination variations, deep shadows, complex surface textures, background noise (such as stains, moss, and dirt), and varying acquisition times. This field realism is essential for developing and testing the robustness of practical automated inspection algorithms.
• **Comprehensive Multi-Task Utility**: The dataset combines multiple annotation types to support several computer vision paradigms simultaneously. By providing pixel-level binary masks, bounding box coordinates, patch-level crack and no-crack classification images, it serves as a unified benchmark for semantic segmentation, instance-style segmentation, object detection, patch classification, and generative image synthesis.
• **Standardized and Reusable Task Protocols**: Concrete400 includes a clearly defined, reproducible training and validation partition for image and mask segmentation experiments (303 training pairs and 76 validation pairs) alongside patch-level classification folders containing 633 crack patches and 869 no-crack patches. This structure supports comparable evaluation of diverse architectural backbones, including crack-oriented CNNs [1], [2], hierarchical vision transformer backbones such as Swin [3], and deformable convolution networks.
• **Realistic Imbalance and Structural Scale Challenges**: With a highly sparse crack pixel density (averaging 3.08% of image area) and crack appearances derived from high resolution field photographs, the dataset represents the scale variations and class imbalance difficulties faced in structural health monitoring. This makes it highly valuable for studying imbalance-aware objectives and supervision strategies such as Focal loss [4], hierarchical crack sample reweighting [5], and Tversky loss [6], multiscale feature fusion, and thin structure attention mechanisms.
• **Enabling Generative Defect Synthesis**: The released images, crack masks, and classification patches open avenues for advanced data augmentation research. Researchers can train generative networks, such as GANs [7] or diffusion models [8], for crack image synthesis workflows, addressing the critical problem of annotation scarcity in civil engineering computer vision.

## 2 Background

Surface cracks are visible indicators of concrete deterioration and are used in condition assessment and maintenance planning. Pixel-level segmentation provides information on crack extent, continuity, branching, and local geometry, but field imagery remains difficult because cracks are thin, irregular, and easily confused with stains or concrete texture. Datasets with dense annotations are thus necessary for the development and evaluation of robust inspection methods.

Existing resources include SDNET2018 for concrete crack recognition [9], the Concrete Crack Segmentation Dataset released by Özgenel [10], and data used to evaluate hierarchical crack extraction networks [1]. More recently, Del Savio et al. provided a dataset of laboratory-classified concrete beams and columns [11], while Hossain et al. released pavement defect imagery for alligator cracks and edge breaks [12]. Pavement crack collections with dense annotation are also widely used as reference points, in particular Crack500 [13] and Cracktree200 [14]. PY-CrackDB combines pixel-level crack masks with image-level crack and no-crack labels for asphalt pavements in Paraguay [15]. Concrete400 complements these resources by providing multi-task annotations for both building surfaces and pavements in Hanoi, Vietnam, capturing a broad range of appearance variations encountered during realistic field inspections.

Further foundational contributions include crack damage detection with convolutional neural networks [16], region-based deep learning for the autonomous detection of multiple structural damage types [17], and broader reviews of structural health monitoring based on deep learning [18]. Concrete400 complements this body of work by providing a multi-task dataset drawn from Vietnamese urban infrastructure under field conditions.

## 3 Data Description

The Concrete400 dataset consists of 379 paired image and mask samples. Each pair has the same base filename, from image_001.jpg to image_379.jpg; representative field images are shown in Fig. 1, patch-level classification examples are shown in Fig. 2, annotated RGB, mask and box examples are shown in Fig. 3, and a mask overlay on the RGB image is shown in Fig. 5. The release archive is organised into three top level data folders, which support five reuse functions as follows:

1. **Segmentation**: semantic crack segmentation and instance-style crack region segmentation. This folder contains Image/ with 379 RGB JPEG photographs resized to 512×512 pixels and Masks/ with 379 corresponding binary crack masks resized to the same dimensions.
2. **Classification**: patch level crack/no-crack image classification. This folder contains two class subdirectories: crack/ with 633 crack patches and no_crack/ with 869 no-crack patches, for a total of 1502 classification patches. Patches were cropped from the source photographs and labelled from the corresponding mask at the time of cropping. The source photograph was therefore known when each label was assigned, but it was not retained in the released file names, which are bare integers, and the exact labelling criterion was not recorded; the provenance of an individual released patch cannot be recovered from the archive. The implications of this limitation are discussed later in this section, alongside Tables 5 and 6.

**Table 1:** Concrete400 dataset characteristics.

Characteristic	Value
Image and mask pairs	379
Annotation types	JPEG RGB images; binary JPEG masks; bounding box annotations for crack regions; PNG bounding box visualisations
Task folder contents	Segmentation: 379 RGB images and 379 masks; Classification: 633 crack patches and 869 no-crack patches; Object Detection: 379 box text files and 379 box visualisation PNGs
Crack region bounding boxes	511 boxes over 379 images (mean 1.3 per image)
Filename range	image_001 to image_379
Image resolution	512×512 pixels
Reference image and mask split	303 training pairs; 76 validation pairs
Binary mask threshold	Pixel value >127 is defined as crack
Mean crack pixel proportion	3.08%
Median crack pixel proportion	2.52%
Crack pixel proportion range	0.40%–15.01%
Released image and mask dimension mismatches	0 pairs; all released segmentation images and masks are 512×512 pixels
Supported task types	Image/patch classification; semantic segmentation; instance-style crack region segmentation; object detection; semi-supervised/self-supervised learning; crack image generation
3. **Object Detection**: crack region bounding box annotations. This folder contains Boxes/ with 379 text annotation files recording crack region bounding boxes, and Boxes_visualizations/ with 379 PNG renderings of the same boxes drawn on their source photograph, one per image, provided for visual inspection rather than for training. The 379 annotation files contain 511 crack region boxes in total, an average of 1.3 boxes per image. The box coordinates are scaled to the released 512×512 RGB images.
4. **Semi-supervised learning**: unlabelled RGB inputs for semi-supervised and self-supervised protocols. Users can treat the released RGB photographs as labelled, partially labelled, or unlabelled inputs for pseudo-labelling, consistency regularisation, teacher-student training, contrastive pretraining, masked-image modelling, or other crack analysis workflows that use few labels.
5. **Generate crack images**: crack image generation and data augmentation. The released crack masks, no-crack classification patches, and RGB photographs can be reused to design crack image synthesis or augmentation workflows, enabling paired or unpaired data generation for downstream segmentation and detection tasks [7], [8].

**Fig. 1:**
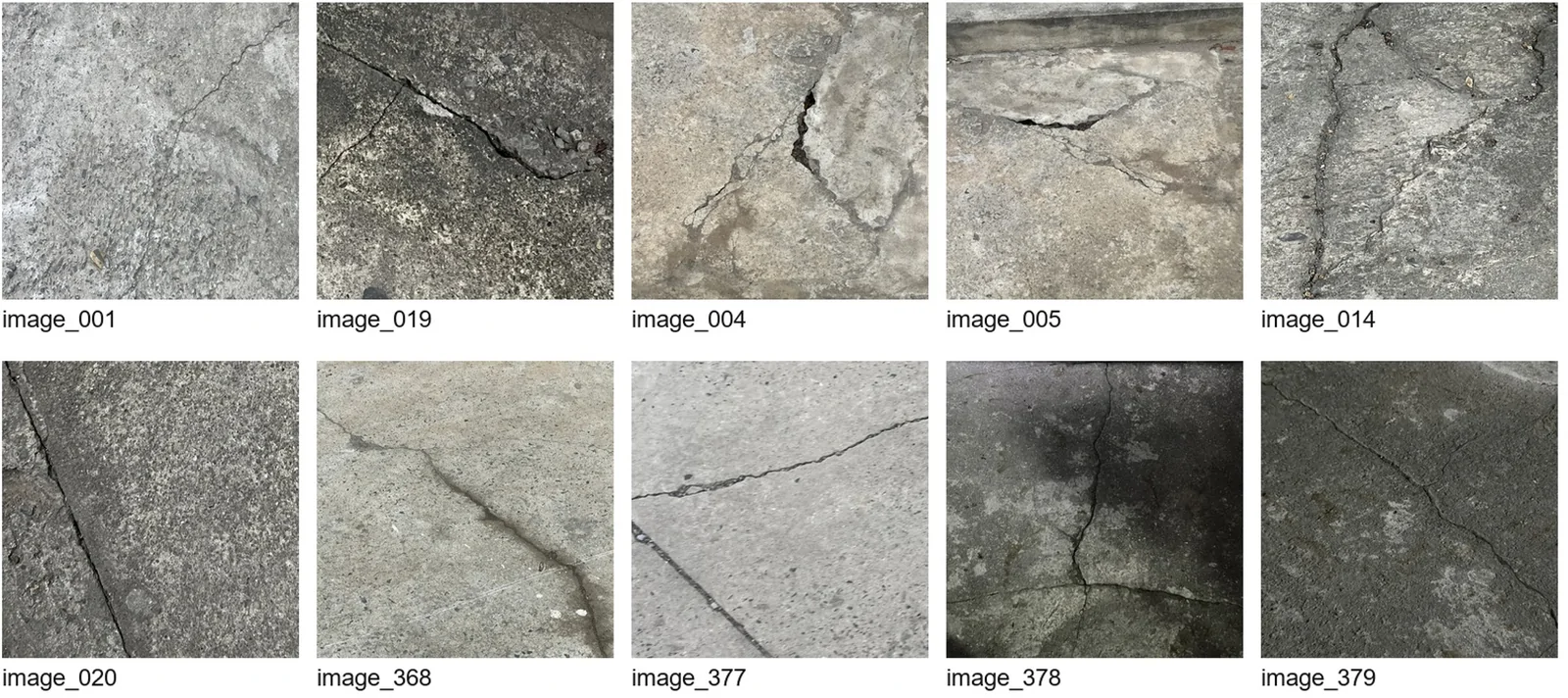
Representative Concrete400 RGB photographs acquired under diverse field conditions, identified by the source image name printed beneath each panel: (a) strong direct daylight and shadow variations (image_001, image_377), (b) complex surface textures and staining (image_019, image_004), (c) wet surface conditions post-rainfall (image_379), (d) background clutter with dirt and moss (image_005, image_014), (e) coarse concrete pavement textures (image_020, image_368), and (f) low-light and night-time acquisition (image_378).

**Fig. 2:**
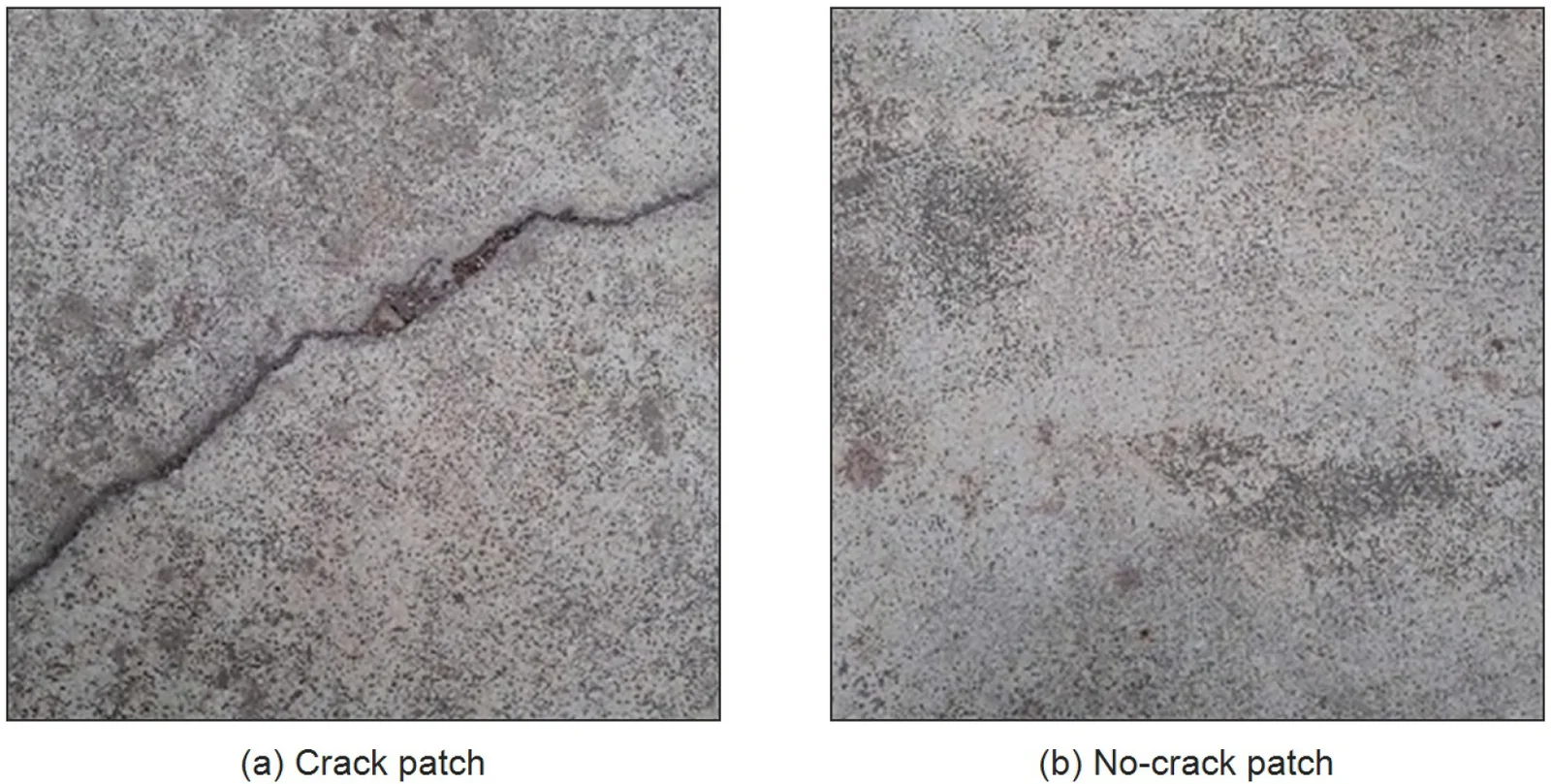
Patch-level examples for classification, showing one patch labelled crack (a) and one labelled no-crack (b) at the released 256×256 resolution.

**Fig. 3:**
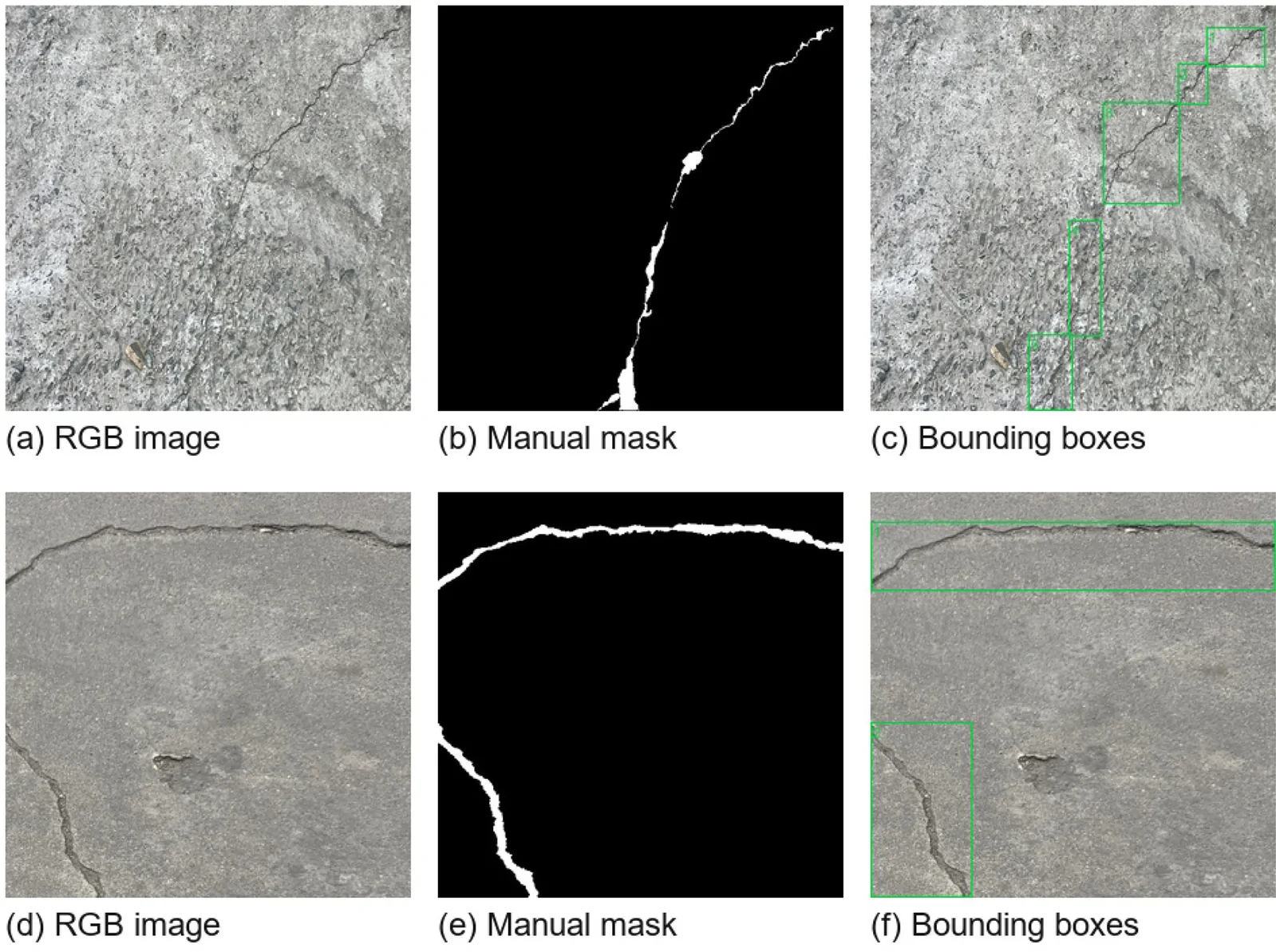
Examples of Concrete400 annotated samples using RGB photographs.

**Fig. 5:**
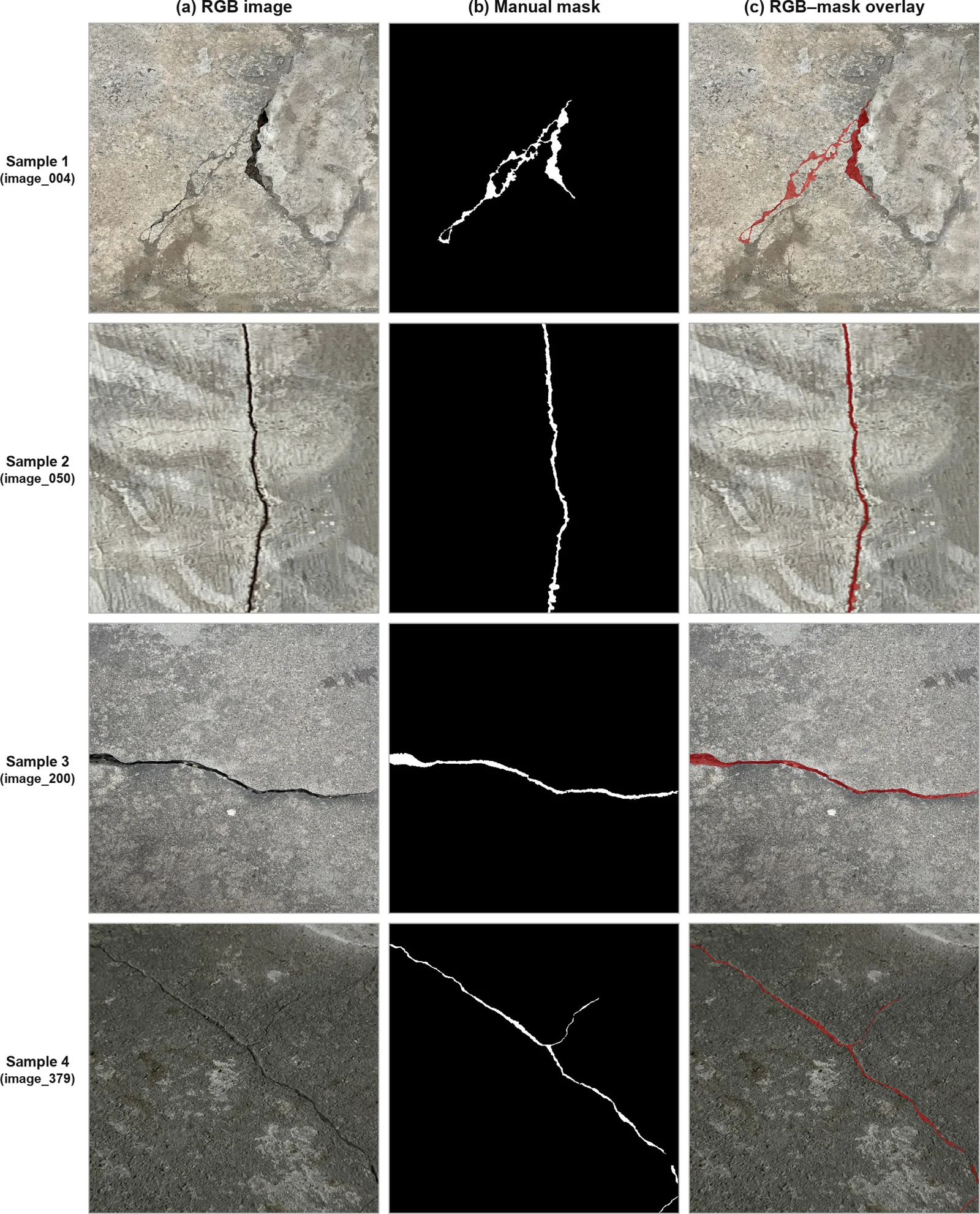
Overlay visualisations of Concrete400 annotations across four representative field samples with diverse crack morphologies and surface conditions: Sample 1 (image_004) irregular crack with branching on a concrete wall, Sample 2 (image_050) vertical linear crack on a smooth concrete wall, Sample 3 (image_200) horizontal crack on dark concrete pavement, and Sample 4 (image_379) branching crack on a wet dark concrete surface. Columns display: (a) source RGB image, (b) manual pixel-level binary mask, and (c) aligned RGB–mask overlay.

Fig. 2 shows the two classification classes as they are released. A crack patch need not be dominated by the crack: the positive class is defined by the presence of crack pixels in the corresponding mask, not by the area they cover, which is why the patch-level task inherits the same sparsity as the segmentation task.

In addition to the image and mask pairs, the release archive includes bounding box annotations for crack regions. These annotations extend the use of Concrete400 beyond dense semantic segmentation. For classification, full images or cropped patches can be assigned crack/non-crack labels according to the presence of crack pixels or boxes. For object detection, each box can be used as a crack region target. For segmentation, the binary masks provide dense semantic labels, while connected crack components, box-constrained regions, or post-processed masks can be used to construct instance style targets when a task requires separating individual crack objects.

The segmentation and object detection components are released at a common 512×512 resolution so that masks and boxes align directly with the RGB images. Table 1 summarises the key characteristics of Concrete400, including dataset size, annotation formats, image resolution, train and validation split, crack pixel distribution, and supported computer vision tasks. These statistics highlight the dataset’s multi-task structure and the realistic class imbalance associated with thin crack regions (Table 2).

**Table 2:** Crack geometry and imaging condition statistics (n=379).

Metric	Mean	Median	Min	Max
Crack width, per-image mean (px)	11.0	9.4	2.3	33.2
Crack width, per-image maximum (px)	26.4	24.0	5.7	99.3
Image brightness, frame mean (0–255)	149.5	150.9	79.3	196.7
Image brightness, within-image s.d. (0–255)	29.8	29.3	5.6	58.2
Crack pixel proportion (%)	3.08	2.52	0.40	15.01

Crack width was computed as twice the Euclidean distance transform sampled along the Zhang–Suen skeleton of each mask, and is reported both as the per-image mean width and as the per-image maximum width; orientation was estimated by principal component analysis of crack pixel coordinates. Folded to 0–$180^{\circ }$, the resulting orientation distribution is close to uniform: the number of images per $30^{\circ }$ bin ranges from 53 to 74 across the six bins. Widths are given in pixels at the released 512×512 resolution. Conversion to physical units was not performed because the original camera resolution and calibration metadata were not retained. In terms of imaging conditions, 99.5% of images fall within a normal ambient brightness range (frame mean 85–200), while 0.5% correspond to low-light or night-time acquisition, consistent with the weather and lighting variability described in Section 4.1. The frame mean is a whole-image average and therefore says little about illumination variation inside a photograph, which is what shadows produce. The within-image standard deviation of brightness is reported for that purpose: it spans 5.6 to 58.2 across the collection, a factor of ten between the most uniformly lit and the most strongly shadowed photograph, and 7.4% of images have at least 5% of their pixels below intensity 64. Both statistics were computed on the released 512×512 images using the mean of the three RGB channels.

Table 3 compares Concrete400 with representative concrete crack datasets discussed in the background section. The comparison follows the compact Data in Brief style and highlights the annotation types and acquisition conditions most relevant for reusing the data in classification, segmentation, and detection studies.

**Table 3:** Dataset comparison.

Dataset	Images	Resolution	Field condition variability	Source	Pixel-wise	Additional annotations	Crack density reported (%)	Supported tasks (#)
SDNET2018 [9]	56,000	256×256	YES	Bridge decks, walls, pavements	NO	YES	–	1
Crack500 [13]	500	∼2560×1440	YES	Pavement surfaces	YES	NO	–	1
Cracktree200 [14]	206	800×600	YES	Pavement surfaces	YES	NO	–	1
PY-CrackDB [15]	569	351×500	YES	Paraguayan roads	YES	YES	–	2
Del Savio et al. [11]	1132	Various	NO	Laboratory beams and columns	NO	YES	–	1
Hossain et al. [12]	12,000	1920×1080	YES	Highway flexible pavements	NO	YES	–	1
Özgenel Dataset [10]	458	4032×3024	YES	Concrete surfaces	YES	NO	–	1
Concrete400	379	512×512	YES	Hanoi concrete surfaces and pavements	YES	YES	3.08	5

A dash in the crack density column means that the value is not reported in the publication releasing that dataset. The column records what each source states rather than a recomputation: crack density depends on the binarisation threshold and on the mask resolution at which it is measured, and these are not documented uniformly across the compared releases, so figures derived under our own conventions would not be comparable with the values their authors would report.

Unlike the compared datasets, which typically support one or two downstream tasks (for example, Del Savio et al. [11] focuses on laboratory-controlled classification, and Hossain et al. [12] provides pavement defect categories without pixel-level masks), Concrete400 is the only dataset in this comparison to provide pixel-wise masks, bounding boxes and patch-level classification labels simultaneously, enabling direct multi-task reuse across five distinct learning paradigms without further data collection or labelling.

The 379 source photographs place Concrete400 within the usual range for specialised crack datasets carrying dense annotation: Table 3 lists Cracktree200 at 206 images, Crack500 at 500 and PY-CrackDB at 569. The released classification folder adds 1502 labelled patches, giving a substantially larger effective sample for patch-level work. The baselines reported in Tables 4–7 indicate that this scale supports meaningful fine-tuning of pretrained architectures, with four segmentation models reaching 0.769–0.787 crack class IoU and three classifiers reaching 0.971–0.978 AUC on the reference validation split. Concrete400 is accordingly intended for fine-tuning, transfer learning and benchmark evaluation rather than for large scale pretraining of foundation models, a boundary also recorded in the Limitations.

**Fig. 4:**
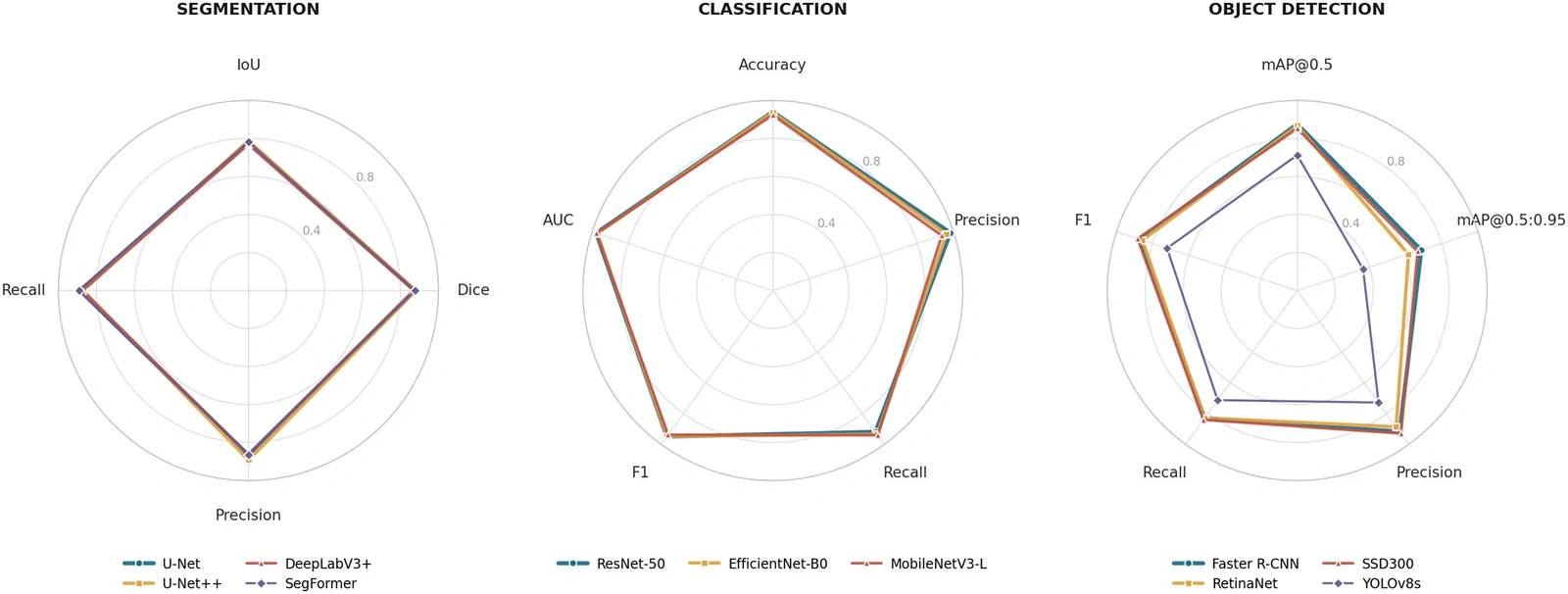
Baseline radar charts for segmentation, classification, and object detection.

**Table 4:** Segmentation baselines on the 76-pair reference validation split (crack class).

Model	IoU	Dice / F1	Precision	Recall	Params (M)
U-Net+ (ResNet-34)	0.7872	0.8810	0.8914	0.8707	26.08
SegFormer (MiT-B2)	0.7819	0.8776	0.8645	0.8912	24.72
U-Net (ResNet-34)	0.7791	0.8758	0.8659	0.8860	24.44
DeepLabV3+ (ResNet-50)	0.7693	0.8696	0.8654	0.8739	26.68

These charts summarise validation behaviour across the three baseline task folders and make the task specific trade-offs easier to compare. Segmentation is mainly differentiated by overlap quality and compactness, classification by the balance between recall and precision, and object detection by the compromise between localisation accuracy, recall, and mAP.

Table 4, Table 5 and 7 report the numerical values underlying Fig. 4, so that the baselines can be compared directly rather than only through the radar plots. All three tables report validation performance on the reference partition defined in Section 4.4: 303 training and 76 validation images, with masks binarised at intensity >127. These baselines are provided to characterise the dataset and are not intended as an optimised state-of-the-art comparison.

The four architectures fall within 0.018 IoU of one another, which is the main observation to draw from this table: once encoder capacity and initialisation are held fixed, the choice of decoder family has little effect on this dataset, and the remaining difficulty lies in the data rather than in the model. Precision and recall trade off in opposite directions across the four models without changing the overall ranking, and the best and worst models differ by 0.011 in Dice.

The patches evaluated in Table 5, and in the regenerated rows of Table 6, were produced from the released 512×512 images and masks under a deterministic rule: the four non-overlapping 256×256 quadrants of each image are cropped, a patch is labelled crack when more than 16 of its mask pixels exceed the binarisation threshold of 127 and no-crack when it contains none, and patches falling between these two cases are discarded as ambiguous. This yields 1494 patches (1037 crack and 457 no-crack), partitioned into 1193 training and 301 validation patches by inheriting the reference image split, so that no source photograph contributes to both subsets. The 16-pixel cut-off corresponds to 0.024% of a 256×256 patch and is deliberately permissive: it is set just above the level at which a patch contains nothing but JPEG ringing along a mask edge, so that patches clipping only the tip of a crack are still labelled crack rather than discarded. Because the rule is deterministic, this patch set can be reconstructed exactly from the released archive.

**Table 5:** Patch-level crack/no-crack classification baselines with patches partitioned by source photograph (301 validation patches).

Model	Accuracy	Precision	Recall	F1	AUC	Params (M)
ResNet-50	0.9402	0.9827	0.9189	0.9497	0.9776	23.51
EfficientNet-B0	0.9369	0.9611	0.9351	0.9479	0.9712	4.01
MobileNetV3-L	0.9236	0.9355	0.9405	0.9380	0.9756	4.20

**Table 6:** Effect of the patch partition protocol on classification scores.

Model	Patch set	Accuracy	F1	AUC
ResNet-50	Regenerated, split by source	0.9402	0.9497	0.9776
ResNet-50	Released, provenance unknown	0.9568	0.9474	0.9973
EfficientNet-B0	Regenerated, split by source	0.9369	0.9479	0.9712
EfficientNet-B0	Released, provenance unknown	0.9867	0.9844	0.9998
MobileNetV3-L	Regenerated, split by source	0.9236	0.9380	0.9756
MobileNetV3-L	Released, provenance unknown	0.9900	0.9882	0.9994

**Table 7:** Crack region object detection baselines on the 76-image validation split (94 boxes).

Model	mAP@0.5	mAP@0.5:0.95	Precision	Recall	F1	Params (M)
Faster R-CNN (R50-FPN)	0.8750	0.6867	0.9176	0.8298	0.8715	41.30
RetinaNet (R50-FPN)	0.8669	0.6159	0.8864	0.8298	0.8571	32.17
SSD300 (VGG16)	0.8527	0.6666	0.9294	0.8404	0.8827	23.75
YOLOv8s	0.7122	0.3647	0.7283	0.7128	0.7204	11.13

The released patch folder is not this set. Its 1502 file names are bare integers drawn from the range 0–4126 with the intervening numbers absent, so the folder is a selected subset of a larger and denser crop enumeration rather than an exhaustive quadrant tiling of the 379 released images, and its class balance differs accordingly: 633 crack and 869 no-crack, against 1037 and 457 for the quadrant tiling above. The selection and labelling rule behind it was not recorded and cannot be recovered from the files, and because the names carry no source image identifier the patches could not be matched back to the released photographs by content. They therefore cannot be partitioned at the level of the source photograph, leakage between the training and validation subsets cannot be excluded, and the two patch sets are not interchangeable. To assess the effect of this limitation, each classifier was additionally trained on the released patches under an otherwise identical recipe; Table 6 places the two protocols side by side.

All three architectures move in the same direction in accuracy and AUC (F1 does not: it falls slightly for ResNet-50, from 0.9497 to 0.9474). Accuracy rises by 1.7, 5.0 and 6.6 percentage points on the released patches and the area under the curve rises to 0.997–1.000. At that level the released partition no longer separates the architectures from one another, a MobileNetV3-L with 4.20 M parameters scoring indistinguishably from a ResNet-50 with 23.51 M. Users of the released patch folder should therefore expect optimistic scores of roughly two to three points of AUC (+2.0, +2.9 and +2.4 points for the three models), and the protocol of Table 5, which partitions patches by source photograph, is the one recommended for reporting.

The segmentation, classification and torchvision detection baselines were initialized with ImageNet-pretrained backbones and fine-tuned using the reference 303/76 image split and a fixed random seed of 42, with AdamW, an initial learning rate of $1\times 10^{-4}$, the pretrained backbone at 0.1× that rate, weight decay 0.01, a cosine schedule decaying to $1\times 10^{-6}$, and gradient clipping at 1.0. Segmentation uses 512×512 inputs, batch size 4, 100 epochs and 10 warm-up epochs, with the objective $L=L_{\mathrm{focal}}+L_{\mathrm{Dice}}+L_{\mathrm{boundary}}$ at equal weights, focal α=0.85 and γ=2.0, and a 3×3 boundary kernel. Classification uses 224×224 inputs, batch size 32, 100 epochs, 3 warm-up epochs and class-weighted cross-entropy. Detection uses 512×512 inputs, batch size 4, 3 warm-up epochs, and 30 epochs for the torchvision detectors and 100 for YOLOv8s. Augmentation comprises horizontal and vertical flips, $90^{\circ }$ rotations, random resized cropping (scale 0.75–1.0), brightness/contrast and gamma jitter, Gaussian noise and blur, elastic deformation and grid distortion, followed by ImageNet channel normalisation. All models were trained on a single NVIDIA GeForce RTX 5060 Laptop GPU under PyTorch 2.11.0, each run taking between 5 and 71 min; checkpoints were selected on the validation metric monitored for each task (crack IoU, AUC and mAP@0.5 respectively), so the reported figures are validation performance at the epoch that scored best on validation rather than an estimate on a held-out test partition, which the reference split does not define. The segmentation models were built with segmentation_models_pytorch 0.5.0 and the first three detectors with torchvision. For every detector, precision, recall and F1 are reported at a single confidence threshold chosen by maximising F1 on the training split and then applied unchanged to validation, so the operating point is never tuned on the data it is reported on, while mAP is computed from the full score range through one shared evaluator.

YOLOv8s is the one exception to this recipe and is reported as a reference point rather than as a matched comparison. It starts from COCO-pretrained rather than ImageNet-pretrained weights and was trained through the Ultralytics pipeline, which supplies its own optimiser, learning-rate schedule and augmentation defaults, for 100 epochs rather than the 30 used for the torchvision detectors; only the input size, batch size and random seed were matched. Its predictions were nevertheless collected on the same validation images and scored through the same evaluator and threshold-selection procedure as the other three, so the metric columns of Table 7 are comparable even though the training configuration is not.

The binary masks include compression and antialiasing values. A documented binarisation rule is therefore required before training and evaluation. The reference protocol defines a pixel as crack when its mask intensity is greater than 127. In the released 512×512 version, each mask already matches the paired RGB image dimensions.

## 4 Experimental Design, Materials and Methods

### 4.1 Image collection

Concrete400 was assembled from high resolution smartphone photographs of cracked building surfaces and concrete pavements in Hanoi, Vietnam. Photographs were acquired under ordinary field conditions and consequently include variations in lighting, shadows, rough backgrounds, surface condition, and acquisition time. The source photographs were standardised to 512×512 pixels for the released segmentation and object detection subsets.

Photographs were captured using the standard rear wide-angle (main) camera at 1× zoom of two Apple smartphones, an iPhone 13 Pro and an iPhone 15 Pro Max, in the default automatic shooting mode without manual exposure or focus control. The camera was held at a distance of approximately 0.3–0.5 m from the crack surface. The shooting angle was kept either perpendicular to the surface or tilted by no more than $15^{\circ }$ from perpendicular. No auxiliary lighting was used; images were captured under available ambient illumination, primarily outdoor daylight, which contributed to the observed illumination and shadow variability. Beyond variation in ambient daylight, the collection spans a range of weather conditions, including overcast and rainy conditions, and includes a small subset of photographs captured under low-light or night-time conditions. A subset of images also depicts wet crack surfaces following rainfall, further contributing to the field realism of the released photographs. Two camera devices were used across the collection; approximately 70% of the photographs were captured with the Apple iPhone 13 Pro and the remaining 30% with the Apple iPhone 15 Pro Max.

### 4.2 Data integrity and preprocessing

All 379 crack masks were manually delineated by a single annotator using Adobe Photoshop, following a standardised step by step protocol. Crack boundaries were traced using the Magnetic Lasso Tool, with edge detection parameters (Width, Contrast, Frequency) adjusted per image to accommodate local crack contrast and texture. Selections were refined using the Select and Mask function (Smooth, Feather and Contrast adjustments) to produce clean crack boundaries. The refined selection was inverted, exported as a new layer mask, and the background was filled with black to produce the final binary mask. All masks were exported as high quality JPEG images in the sRGB colour space with metadata stripped. To ensure annotation quality, all 379 crack masks were subsequently reviewed by a second member of the research team, who visually verified each mask against its source photograph and flagged inaccuracies for correction by the original annotator. This workflow of annotation followed by verification was applied to the entire dataset (379/379 images, 100% coverage).

Image and mask filenames were matched to check pair integrity; all 379 images have a corresponding mask. During standardisation, RGB images were resized directly to 512×512 pixels, masks were resized to 512×512 pixels with nearest-neighbour interpolation, and object detection bounding box coordinates were scaled to the same 512×512 coordinate system. Therefore, the released segmentation images, masks, and detection boxes are spatially aligned without requiring additional resizing.

The accompanying training recipe uses a fixed random seed of 42 and optional geometric augmentation: horizontal and vertical flips, $90^{\circ }$ rotations, random resized crops, elastic deformation, and grid distortion. These augmentations are not part of the raw dataset and should be reported separately by users who adopt them.

### 4.3 Learning protocols supported by the dataset

Concrete400 can be used under several supervision regimes. In fully supervised learning, masks are used for semantic segmentation, boxes for object detection, and derived image or patch labels for classification. If the objective is instance-style crack region segmentation, connected components or box-constrained mask regions can be used as object level targets.

The dataset can also support weakly supervised and semi-supervised settings. Bounding boxes can serve as weaker annotations than full masks for box-supervised segmentation or segmentation pipelines that detect first. A subset of masks can be treated as labelled data, while the remaining RGB images are used with pseudo-labelling, consistency regularisation, or teacher-student training. In unsupervised or self-supervised settings, the RGB images can be used without labels for representation learning, contrastive pretraining, masked-image modelling, crack discovery by clustering, or crack localisation treated as anomaly detection; the released masks and boxes can then be reserved for downstream evaluation.

### 4.4 Train and validation split and recommended reporting

The reference split sorts base filenames lexicographically and assigns the first 80% of pairs to training and the remainder to validation: image_001 to image_303 (303 pairs) and image_304 to image_379 (76 pairs). The partition is therefore a deterministic benchmark split. It is not stratified by imaging condition, and users who require evaluation stratified by illumination, surface condition or acquisition time should define their own partition. Importantly, the validation set was not used for gradient-based parameter updates or model training; validation metrics were used only for performance monitoring and checkpoint selection on the reference partition. Studies using another partition should state their split generation method, random seed, input resolution, mask threshold, annotation type, preprocessing sequence, and loss function, especially when using imbalance-aware objectives or supervision strategies such as Focal loss, hierarchical crack sample reweighting, or Tversky loss [4], [5], [6]. For segmentation, precision, recall, F1-score, crack class intersection over union, and mean IoU should be reported. For object detection, box level precision, recall, average precision, and mean average precision can be reported. For classification, accuracy, precision, recall, F1-score, and area under the receiver operating characteristic curve are appropriate, especially when crack and non-crack crops are imbalanced.

## Limitations

Concrete400 is focused on concrete surfaces in Hanoi, Vietnam, so models should be validated before use in other regions, materials, or imaging conditions. Masks are stored as JPEG files, and the dataset does not include device metadata, geographic coordinates, crack width measurements in physical units, or material history records. All photographs were captured with two smartphone models from the same manufacturer (Apple iPhone 13 Pro and iPhone 15 Pro Max) under a mix of ambient outdoor conditions, so the dataset covers two sensor generations but does not span different manufacturers or non-smartphone imaging systems; although the collection includes variability in weather (including rain), ambient lighting (including some night-time and low-light images), and surface wetness, these attributes were not systematically logged or labelled per image at the time of acquisition. The same applies to the capture device: the approximate proportion contributed by each of the two devices is known (about 70% and 30%), but the device used for any individual image is not recorded, so users requiring evaluation stratified by device or by imaging condition would need to perform this labelling themselves. Crack masks were produced by a single annotator and subsequently verified for accuracy by a second team member across the entire dataset (379/379 images); because this verification involved flagging errors on existing masks rather than independent parallel annotation, a formal inter-annotator agreement statistic could not be computed. The absolute scale of the dataset (379 image and mask pairs) is suited to fine-tuning and benchmark evaluation rather than to large scale pretraining of foundation models. Pavement versus building surface origin is not currently labelled per image; this is noted as a direction for future extension of the dataset. Crack morphology/type is also not formally labelled per image; although representative linear and branching crack morphologies are illustrated in Figs. 1 and 5, categorical crack-type annotations are not included in the current release.

## Ethics Statement

The dataset was created for infrastructure surface analysis and does not involve human participants, animals, social media data, or personal identifiers. All photographs were reviewed before public release, and no identifiable personal information was present. Therefore, formal ethical approval and informed consent were not required.

## CRediT authorship contribution statement

**Le Hoai Nam:** Conceptualization, Investigation, Writing – original draft. **Nguyen Ngoc Lan:** Conceptualization, Investigation, Writing – original draft. **Vu Manh Trung:** Methodology, Software, Validation, Writing – review & editing. **Nguyen Nam Son:** Data curation, Formal analysis, Visualization. **Vu Quang Tuyen:** Formal analysis, Validation, Data curation. **Tran Ngoc Hoa:** Supervision, Project administration, Writing – review & editing.

## Data Availability

The Concrete400 dataset is publicly available on Zenodo (https://doi.org/10.5281/zenodo.21193945). That record contains the segmentation, object detection and classification folders described in Section 2. The provenance-tracked patch set evaluated in Table 5 and in the regenerated rows of Table 6 is not distributed as a separate archive: it is derived from the released images and masks by the deterministic cropping and labelling rule stated in Section 3, and can therefore be reconstructed exactly from the Zenodo record. Concrete400 (Original data; Zenodo).
